# Supplementary material for: Chromosomally unstable tumor cells specifically require KIF18A for proliferation
Source: Nat Commun. 2021 Feb 22;12:1213. doi: 10.1038/s41467-021-21447-2 (PMC7900194; doi:10.1038/s41467-021-21447-2)
Supplement: Supplementary file 1 — Supplementary Information [file 41467_2021_21447_MOESM1_ESM.pdf]

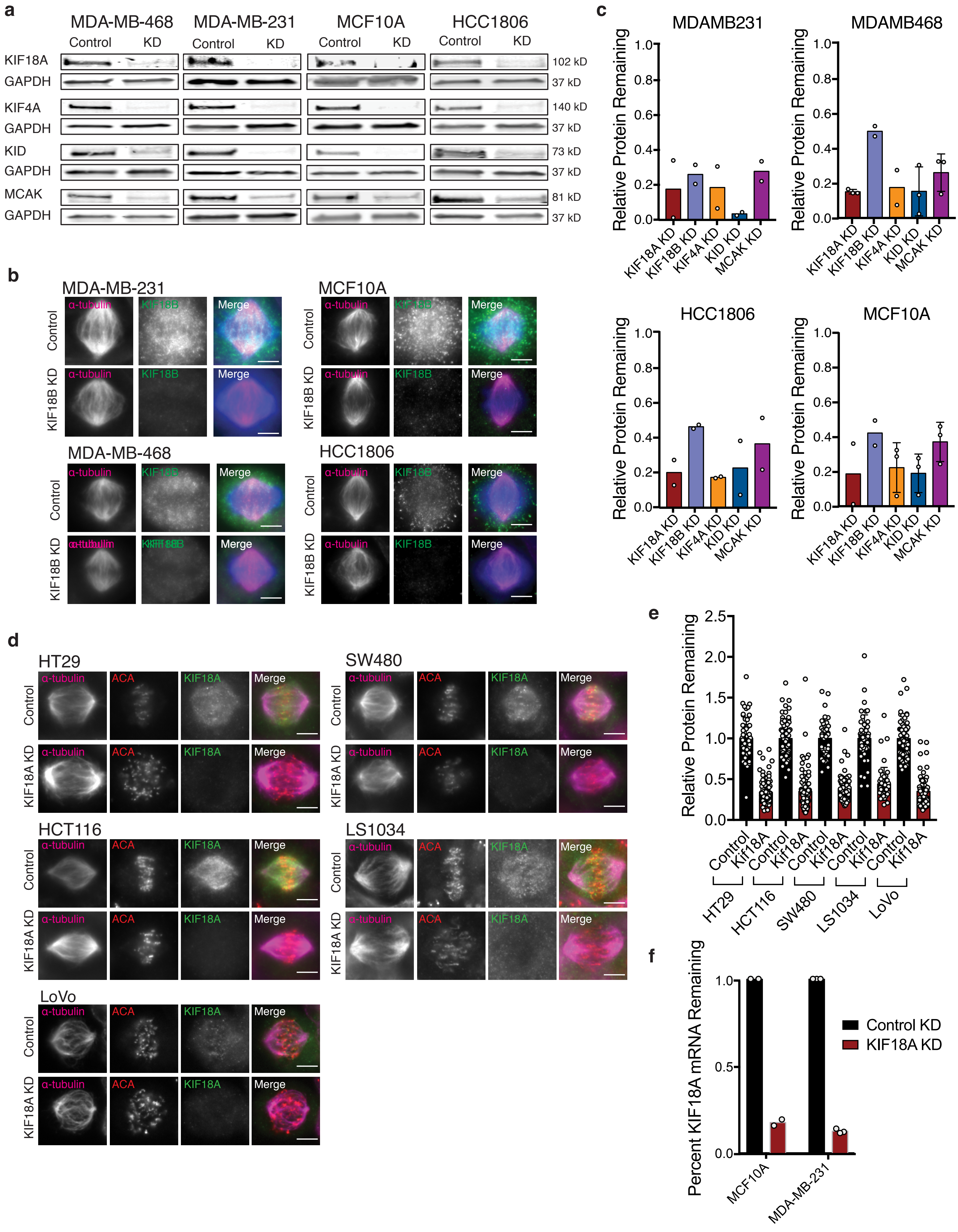

a
MDA-MB-468
MDA-MB-231
MCF10A
HCC1806
Control
KD
KIF18A
GAPDH
KIF4A
KID
MCAK
102 kD
37 kD
140 kD
73 kD
81 kD
b
MDA-MB-231
MCF10A
MDA-MB-468
HCC1806
Control
KIF18B KD
α-tubulin
KIF18B
Merge
c
MDAMB231
MDAMB468
HCC1806
MCF10A
Relative Protein Remaining
KIF18A KD
KIF18B KD
KIF4A KD
KID KD
MCAK KD
d
HT29
SW480
HCT116
LS1034
LoVo
Control
KIF18A KD
α-tubulin
ACA
KIF18A
Merge
e
Relative Protein Remaining
Control
Kif18A
HT29
HCT116
SW480
LS1034
LoVo
f
Percent KIF18A mRNA Remaining
Control KD
KIF18A KD
MCF10A
MDA-MB-231

Supplementary Figure 1. Kinesins are effectively depleted by siRNA in breast and colorectal cell lines.

a Representative Western blots (from 2 or 3 independent experiments, see below) of the indicated proteins following siRNA knockdown (KD) of kinesins in TNBC and diploid breast epithelial cells. Full scans of blots are available in Source Data. **b** Representative immunofluorescence images (from 2 independent experiments) of KIF18B after siRNA treatment in mitotic TNBC and diploid breast epithelial cells. Scale bar is 10 microns. **c** Quantification of kinesin knockdowns in TNBC and diploid breast epithelial cells, measured via Western blot or immunofluorescence, normalized to control siRNA condition. n = 2 (MDA-MB-231- all conditions), 2 (MDA-MB-468- KIF18B KD, KIF4A KD), 3 (MDA-MB-468- KIF18A KD, KID KD, MCAK KD), 2 (HCC1806- all conditions), 2 (MCF10A- KIF18A KD, KIF18B KD), and 3 (MCF10A- KIF4A KD, KID KD, MCAK KD) independent biological replicates. **d** Representative immunofluorescence images (from 2 independent experiments) of KIF18A following siRNA-mediated knockdown in CRC cell lines. Scale bar is 10 microns. **e** Quantification of KIF18A knockdown in CRC cell lines measured via immunofluorescence and normalized to control. n (control KD/ KIF18A KD) = 59/96 (HT29), 62/78 (HCT116), 36/80 (SW480), 47/46 (LS1034), and 58/75 (LoVo) cells from two independent experiments. **f** qRT-PCR measurements of KIF18A mRNA levels after siRNA-mediated knockdown in MCF10A and MDA-MB-231 cells. n = 2 (MCF10A) and 3 (MDA-MB-231) independent biological replicates. All graphs show mean values and individual data points. Error bars represent SD.

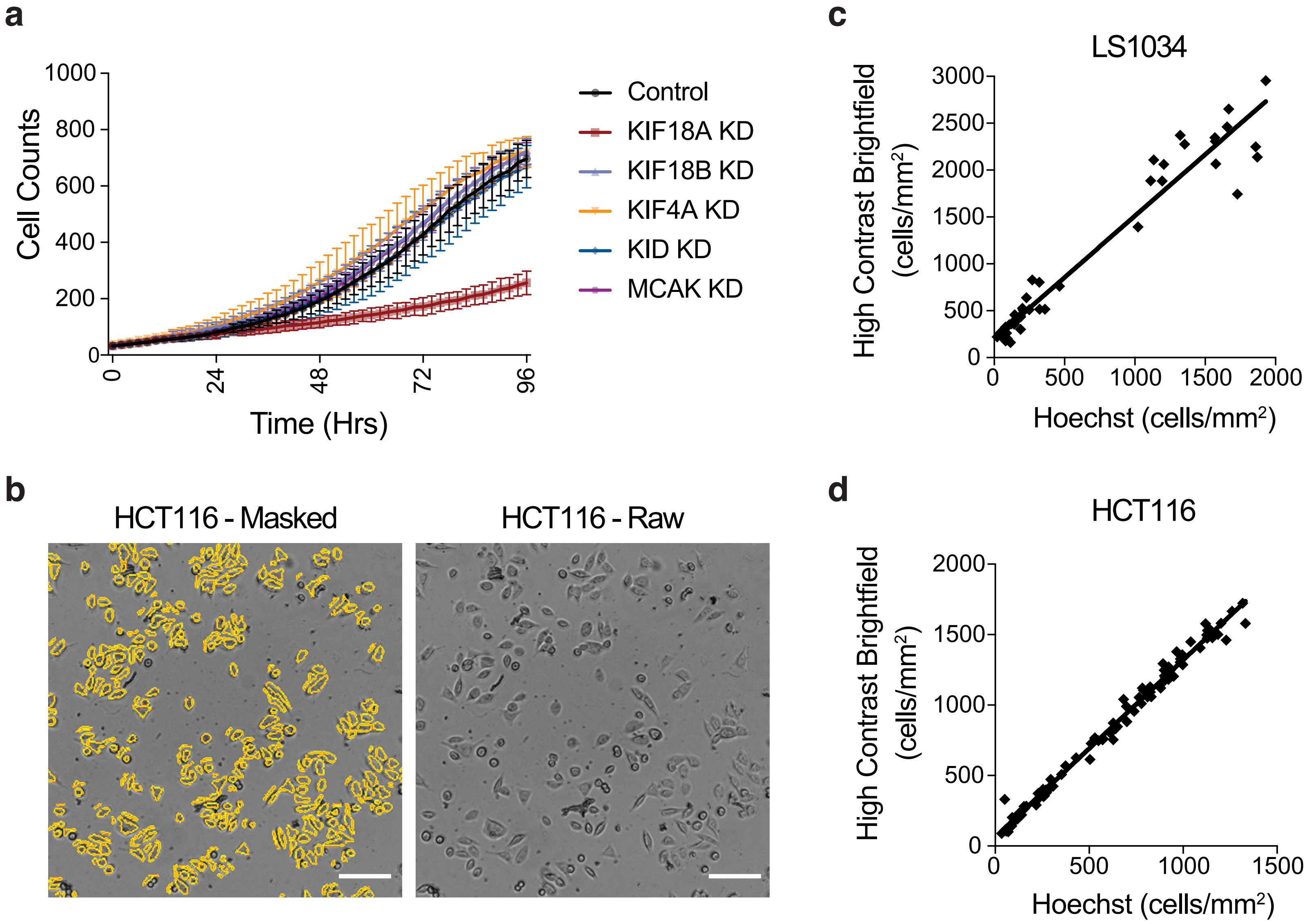

Supplementary Figure 2. Kinetic cell proliferation assay validation.
a Example trace of MDA-MD-231 cell density (cells/mm2) following treatment with the indicated siRNAs as a function of time over 96 hours. n = 10 wells of cells from a single experiment per condition. Error bars represent SD. **b** Representative images (from 5 independent experiments) of HCT116 cells showing the masks created for automated cell counting. Scale bar is 100 μm. **c-d** Scatterplots of automated (**c**) LS1034 and (**d**) HCT116 cell counts using high-contrast brightfield microscopy as a function of cell counts of the same fields using a nuclear dye (Hoechst). Linear correlation indicates consistency in automated cell counting across different cell densities. Data are from a single experiment.

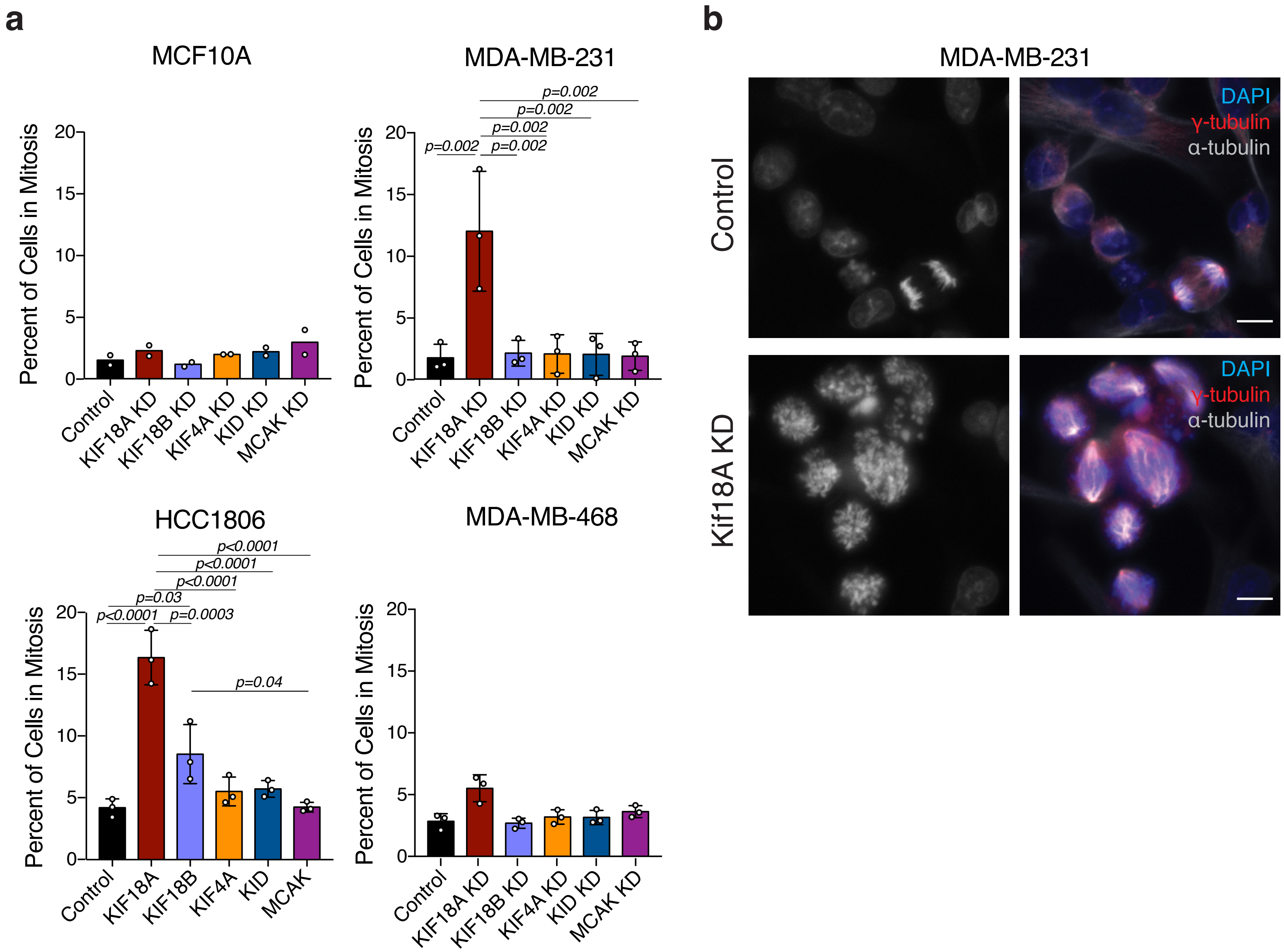

Supplementary Figure 3. KIF18A KD increases the mitotic index of TNBC but not diploid breast epithelial cells.

a Percent of cells in mitosis, as determined from fixed cell images, 48 hours after siRNA-mediated knockdown (KD) of the specified kinesins. n = 3 (MDA-MB-231, HCC1806, MDA-MB-468) or 2 (MCF10A) independent biological samples. Data were analyzed via one-way ANOVA with post-hoc Tukey's test for multiple comparisons. p-values < 0.05 are displayed on plots. All graphs show mean and individual data points. Error bars represent SD. **b** Representative images (from 3 independent experiments) of MDA-MB-231 cells treated with either control or KIF18A siRNA. DNA (DAPI, blue), microtubules (α-tubulin, white), and centrosomes (γ-tubulin, red) are labeled. Scale bar is 10 microns.

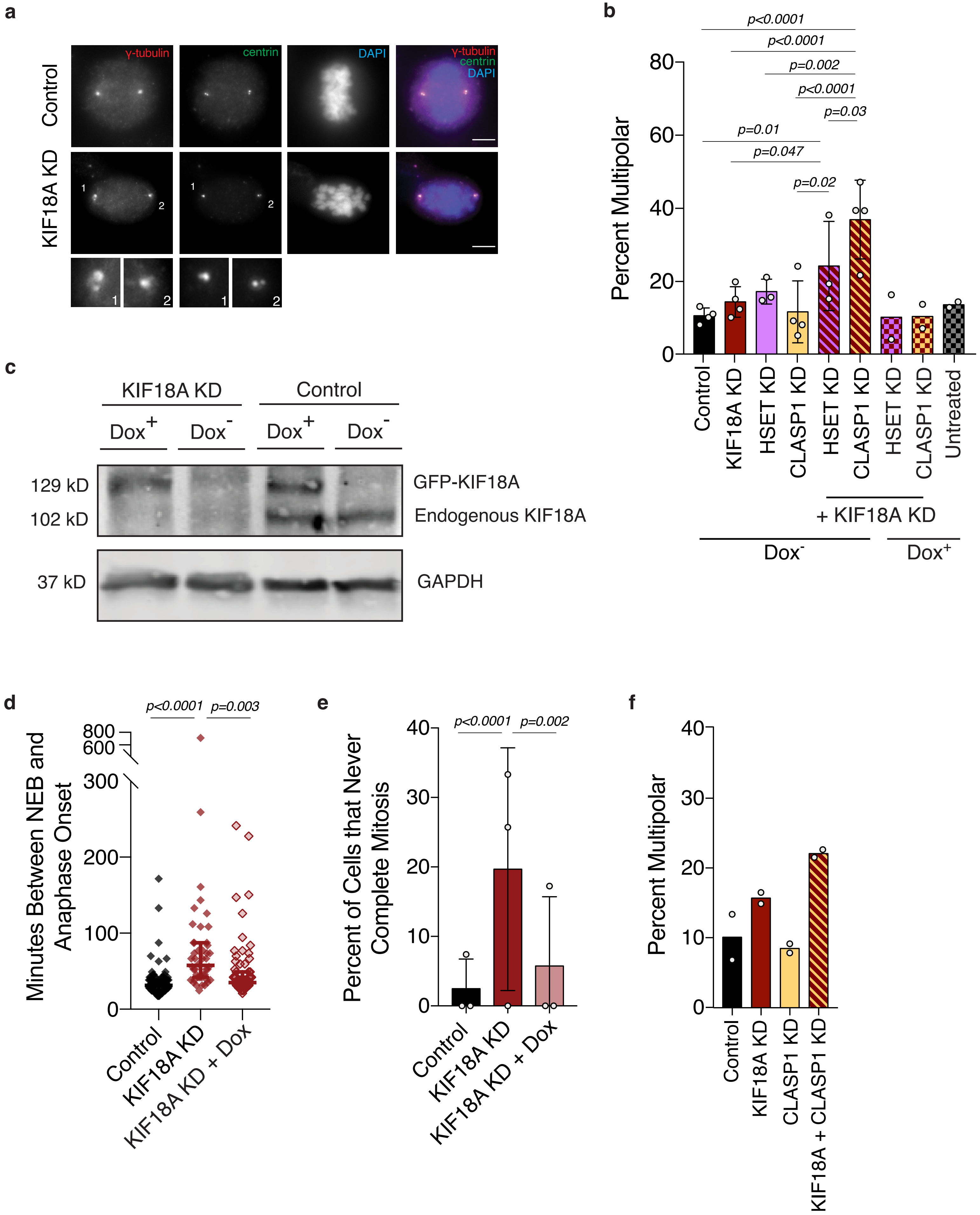

a
Control
KIF18A KD
γ-tubulin
centrin
DAPI
1
2
b
p<0.0001
p<0.0001
p=0.002
p<0.0001
p=0.03
p=0.01
p=0.047
p=0.02
Percent Multipolar
0
20
40
60
80
Control
KIF18A KD
HSET KD
CLASP1 KD
HSET KD
CLASP1 KD
HSET KD
CLASP1 KD
Untreated
+ KIF18A KD
Dox-
Dox+
c
KIF18A KD
Control
Dox+
Dox-
Dox+
Dox-
129 kD
102 kD
37 kD
GFP-KIF18A
Endogenous KIF18A
GAPDH
d
p<0.0001
p=0.003
Minutes Between NEB and Anaphase Onset
0
100
200
300
600
800
Control
KIF18A KD
KIF18A KD + Dox
e
p<0.0001
p=0.002
Percent of Cells that Never Complete Mitosis
0
10
20
30
40
Control
KIF18A KD
KIF18A KD + Dox
f
Percent Multipolar
0
10
20
30
40
Control
KIF18A KD
CLASP1 KD
KIF18A + CLASP1 KD

Supplementary Figure 4. Co-depletion of KIF18A and CLASP1 or HSET lead to synergistic increases in multipolar spindle formation

a Representative images (from 4 independent experiments) of HeLa Kyoto cells treated with either control or KIF18A siRNA. DNA (DAPI, blue), centrosomes (γ-tubulin, red), and centrioles (Centrin-1, green) are labeled. Insets show enlarged images of the numbered spindle poles in the KIF18A KD cell. Scale bar is 10 microns. **b** Plot showing the percentage of HeLa Kyoto cells with multipolar spindles following treatment with the indicated siRNAs, with or without induction of GFP-KIF18A via doxycycline. n (number of cells/ number of independent experiments) = 86/4 (control KD), 133/4 (KIF18A KD), 79/3 (HSET KD), 103/4 (CLASP1 KD), 125/3 (KIF18A + HSET KD), 102/4 (KIF18A + CLASP1 KD), 62/2 (KIF18A + HSET KD + dox), 101/2 (KIF18A + CLASP1 + dox), and 82/2 (untreated + dox). Data were analyzed via two-sided Chi-square test. **c** Western blot of KIF18A and GAPDH in cells treated with KIF18A or control siRNA with or without the addition of doxycycline to induce expression of GFP-KIF18A. Full scan of blot is available in Source Data. **d** Time between nuclear envelope breakdown (NEB) and anaphase onset for HeLa Kyoto cells treated with control or KIF18A siRNA with or without the addition of doxycycline. n = 114 (control KD), 46 (KIF18A KD), and 68 cells (KIF18A KD + dox) from 3 independent experiments. Data were analyzed via a one-way ANOVA with post-hoc Tukey's test. **e** Percent of HeLa Kyoto cells that fail to complete mitosis after treatment with either control or KIF18A siRNA with or without doxycycline. n = 116 (control KD), 63 (KIF18A KD), and 73 cells (KIF18A KD + dox) from 3 independent experiments. Data were analyzed via two-sided Chi-square test. **f** Percent of SW480 cells with multipolar spindles following treatment with the indicated siRNAs. n = 119 (control KD), 148 (KIF18A KD), 136 (CLASP1 KD), and 155 cells (KIF18A + CLASP1 KD) from two independent experiments. All graphs show mean and individual data points. Error bars represent SD. p-values < 0.05 from the indicated statistical tests are displayed on plots.

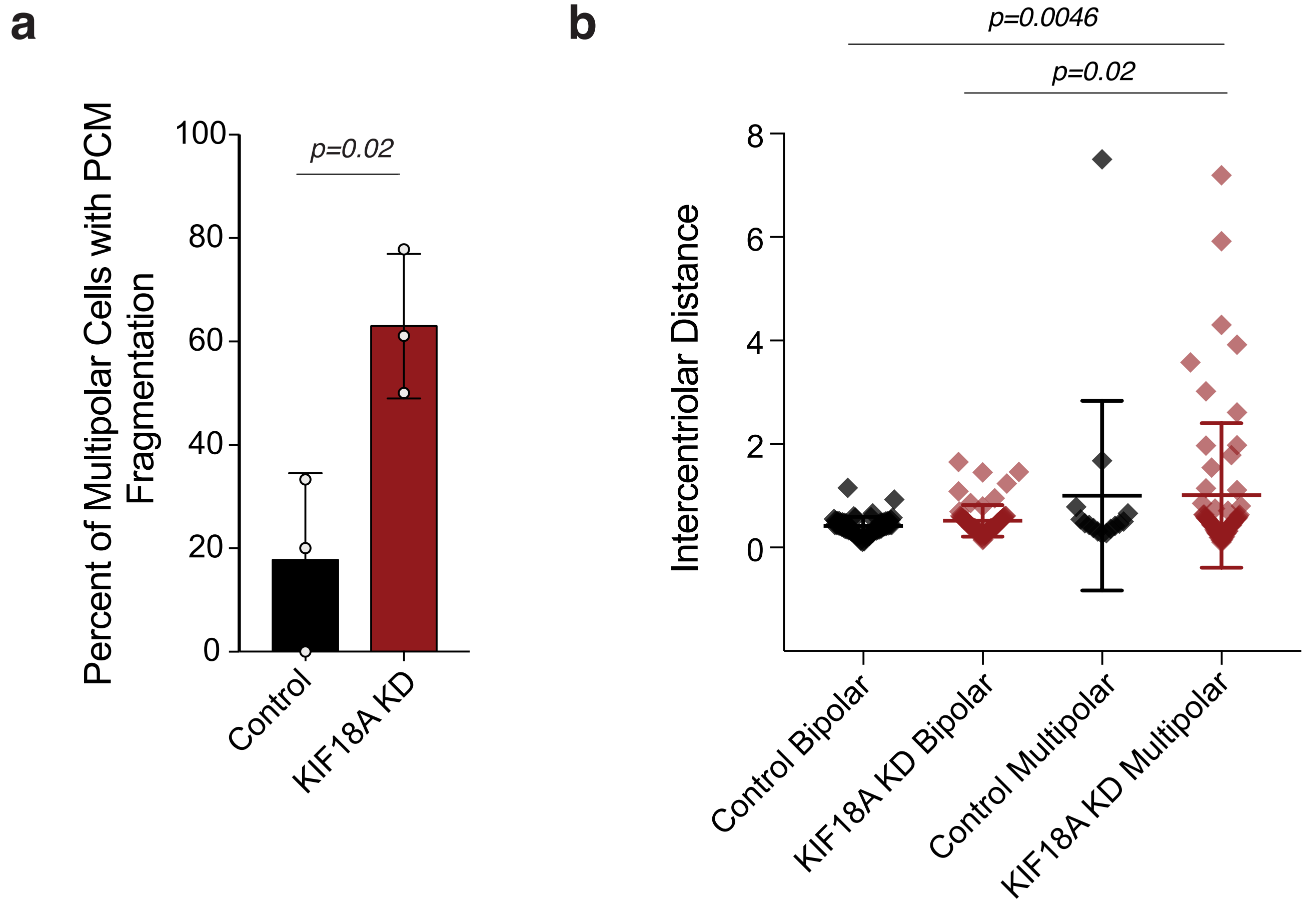

Supplementary Figure 5. Loss of KIF18A causes centrosome fragmentation in HT29 cells
a Percent of HT29 cells with fragmented pericentriolar material (PCM), as indicated by the presence of -tubulin puncta lacking centrin-1. n = 3 independent biological samples. Data were analyzed by unpaired, two-tailed t-test. **b** Intercentriolar distance measurements (in microns) for HT29 cells in each of the indicated categories. n = 58 (control KD, bipolar), 63 (KIF18A KD, bipolar), 15 (control KD, multipolar), and 60 cells (KIF18A KD, multipolar) from three independent experiments. Data were analyzed via a one-way ANOVA with post-hoc Tukey's test. All graphs show mean +/- SD and individual data points. p-values < 0.05 are displayed.

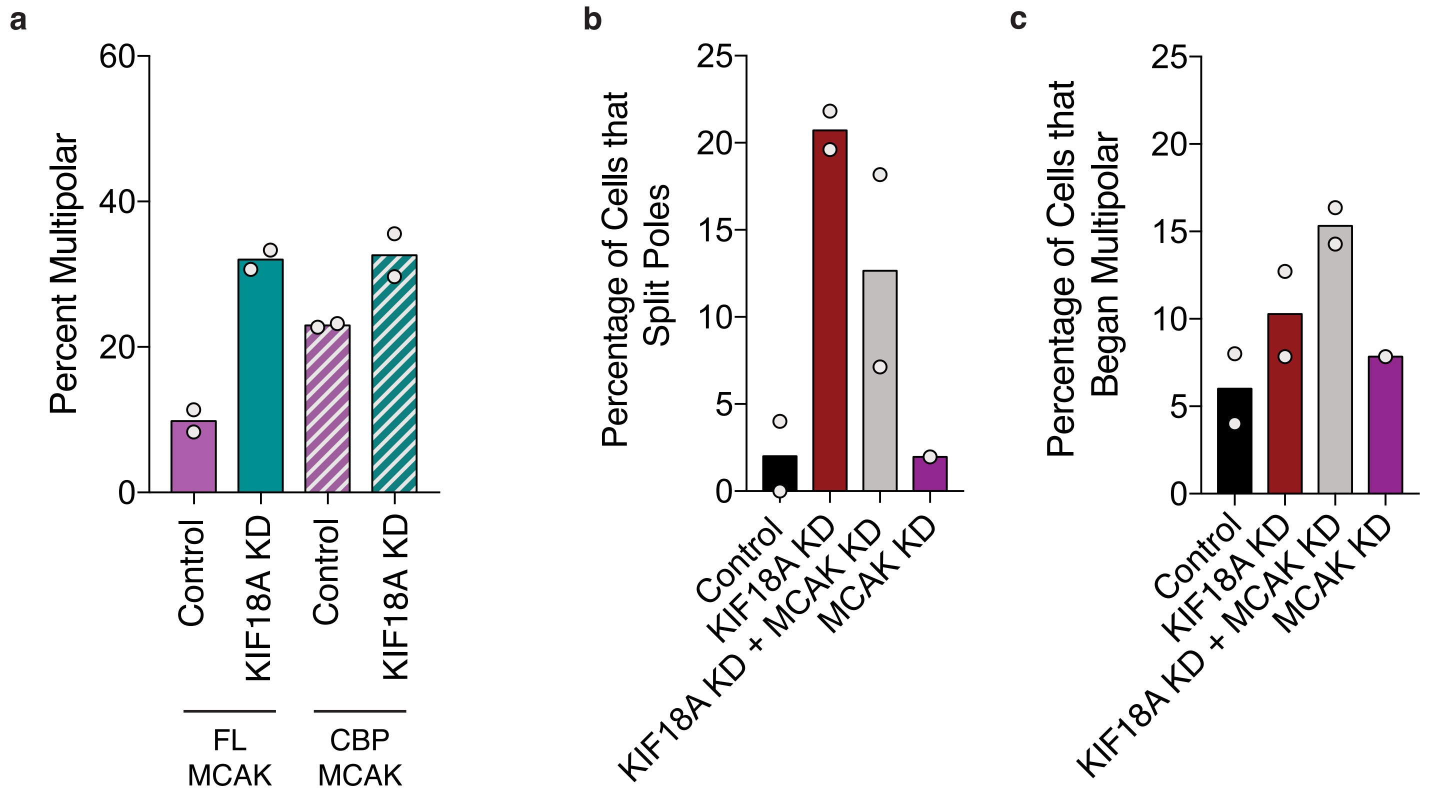

Supplementary Figure 6. Proliferation and multipolar spindle defects caused by KIF18A KD are sensitive to changes in KIF2C/MCAK activity.

a Percent of MDA-MB-231 cells with multipolar spindles following transfection with the indicated siRNAs and mCh-full-length-MCAK (FL MCAK) or mCh-CPB-MCAK (CPB MCAK), which localizes to centromeres via the CENP-B DNA-binding domain. n = 102 (control KD + FL MCAK), 202 (KIF18A KD + FL MCAK), 113 (control KD + CPB MCAK), and 187 cells (KIF18A KD + CPB MCAK) from two independent experiments. **b-c** Percent of live, siR-tubulin labeled MDA-MB-231 cells that (**b**) split poles or (**c**) entered mitosis with more than two spindle poles after treatment with the indicated siRNAs. n (number of cells/ number of independent experiments) = 100/2 (control KD), 106/2 (KIF18A KD), 111/2 (KIF18A + MCAK KD), and 51/1 (MCAK KD). All graphs show mean and individual data points.

Primers	Sequence (5'-3')
**For generation of GFP-KIF18A inducible cell line**	
**GFP-KIF18A- Fwd**	tttagtgaaccgtcagatcgCGCTAGCGCTACCGGTCGCC
**GFP-KIF18A- Rev**	catacattatacgaagttatTACAAGAAAGCTGGGTCGGCGC
**pEM791- Fwd**	GCCGACCCAGCTTTCTTGTAataacttcgtataatgtataatgtatgctatacg
**pEM791- Rev**	GGCGACCGGTAGCGCTAGCGcgatctgacggttcactaaa

Supplementary Table 1- Primers used in this study.
Uppercase letters indicate target specific bases for GFP-KIF18A backbone
Lowercase letters indicate target specific bases for pEM791 backbone
