# Supplementary material for: Chromosomally unstable tumor cells specifically require KIF18A for proliferation
Source: Nat Commun. 2021 Feb 22;12:1213. doi: 10.1038/s41467-021-21447-2 (PMC7900194; doi:10.1038/s41467-021-21447-2)
Supplement: Supplementary file 3 — Description of Additional Supplementary Files [file 41467_2021_21447_MOESM3_ESM.pdf]

Description of Additional Supplementary Files

File Name: Supplementary Movie 1.

Description: Representative time-lapse movie (from three independent experiments) of bipolar division in a siR-tubulin labeled MDA-MB-231 cell treated with control siRNA. Images were acquired every 2 min and are played back at 7 frames per second.

File Name: Supplementary Movie 2.

Description: Representative time-lapse movie (from three independent experiments) of spindle pole splitting in a siR-tubulin labeled MDA-MB-231 cell treated with KIF18A siRNA. Images were acquired every 2 min and are played back at 7 frames per second.

File Name: Supplementary Movie 3.

Description: Representative time-lapse movie (from three independent experiments) of a siR-tubulin labeled MDA-MB-231 cell treated with KIF18A siRNA entering mitosis with multiple spindle poles. Images were acquired every 2 min and are played back at 7 frames per second.

File Name: Supplementary Movie 4.

Description: Representative time-lapse movie (from two independent experiments) of spindle pole splitting in a siR-tubulin labeled MDA-MB-231 cell treated with KIF18A and MAD2 siRNAs. Images were acquired every 2 min and are played back at 7 frames per second.

File Name: Supplementary Movie 5.

Description: Representative time-lapse movie (from two independent experiments) of centrosome fragmentation in a monopolar MDA-MB-231 cell expressing mRFP-pericentrin after treatment with KIF18A siRNA and 20 μM monastrol. Images were acquired every 2 min and are played back at 7 frames per second.

File Name: Supplementary Movie 6.

Description: Representative time-lapse movie (from three independent experiments) of spindle pole splitting in a siR-tubulin labeled MDA-MB-231 cell treated with KIF18A siRNA and 500 nM UMK57. Images were acquired every 2 min and are played back at 7 frames per second.
