# Supplementary material for: Chromosomally unstable tumor cells specifically require KIF18A for proliferation
Source: Nat Commun. 2021 Feb 22;12:1213. doi: 10.1038/s41467-021-21447-2 (PMC7900194; doi:10.1038/s41467-021-21447-2)
Supplement: Supplementary file 2 — Peer Review File [file 41467_2021_21447_MOESM2_ESM.pdf]

Editorial Note: This manuscript has been previously reviewed at another journal that is not operating a transparent peer review scheme. The manuscript was considered suitable for publication without further review at Nature Communications.
